# [Retracted] Effects of RUNX3 mediated Notch signaling pathway on biological characteristics of colorectal cancer cells

**DOI:** 10.3892/ijo.2024.5679

**Published:** 2024-08-05

**Authors:** Hang Li, Dan Li, Ning Meng

Int J Oncol 50: 2059-2068, 2017; DOI: 10.3892/ijo.2017.3988

Following the publication of this paper, it was drawn to the Editors' attention by a concerned reader that certain of the cell apoptotic data shown in Fig. 5A on p. 2065, and the Transwell migration and invasion assay data shown in Fig. 6A and C on p. 2066 were strikingly similar to data appearing in different form in other articles written by different authors at different research institutes that had either already been published elsewhere prior to the submission of this paper to *International Journal of Oncology*, or were submitted for publication at around the same time. Given that the abovementioned data had already apparently been published previously, the Editor of *International Journal of Oncology* has decided that this paper should be retracted from the Journal. The authors were asked for an explanation to account for these concerns, but the Editorial Office did not receive a reply. The Editor apologizes to the readership for any inconvenience caused.

